# The role of Atlantic overturning circulation in the recent decline of Atlantic major hurricane frequency

**DOI:** 10.1038/s41467-017-01377-8

**Published:** 2017-11-22

**Authors:** Xiaoqin Yan, Rong Zhang, Thomas R. Knutson

**Affiliations:** 1 The Program in Atmospheric and Oceanic Sciences, Princeton University, Princeton, NJ 08540 USA; 2 NOAA/GFDL, Princeton, NJ 08540 USA

## Abstract

Observed Atlantic major hurricane frequency has exhibited pronounced multidecadal variability since the 1940s. However, the cause of this variability is debated. Using observations and a coupled earth system model (GFDL-ESM2G), here we show that the decline of the Atlantic major hurricane frequency during 2005–2015 is associated with a weakening of the Atlantic Meridional Overturning Circulation (AMOC) inferred from ocean observations. Directly observed North Atlantic sulfate aerosol optical depth has not increased (but shows a modest decline) over this period, suggesting the decline of the Atlantic major hurricane frequency during 2005–2015 is not likely due to recent changes in anthropogenic sulfate aerosols. Instead, we find coherent multidecadal variations involving the inferred AMOC and Atlantic major hurricane frequency, along with indices of Atlantic Multidecadal Variability and inverted vertical wind shear. Our results provide evidence for an important role of the AMOC in the recent decline of Atlantic major hurricane frequency.

## Abstract (web summary)

The reason for the decline in frequency of Atlantic major hurricanes since 2005 is still debated. Here the authors combine observations with model simulations to show that this decline is largely due to changes in the Atlantic ocean meridional overturning circulation.

## Introduction

Atlantic major hurricane activity has caused substantial damage to coastal settlements and infrastructure^1^. Observed Atlantic major hurricane frequency exhibits prominent multidecadal variability^2–5^, with high values in the 1950s, a rapid decline in the 1960’s and an abrupt increase in the 1990s. The most recent above-normal period of activity peaked around 2005. There were a remarkable seven Atlantic major hurricanes in 2005, including Hurricane Katrina (category 5) with losses of more than $100 billion. Observed multidecadal variability in Atlantic hurricane frequency was found to be well-correlated with low-pass filtered detrended area-averaged North Atlantic sea surface temperature (SST) anomalies, i.e., the so-called Atlantic multidecadal variability (AMV) Index^6, 7^, in previous studies^4, 8–11^. However, there is currently no consensus on the mechanism causing the AMV and the linkage between the AMV and the Atlantic meridional overturning circulation (AMOC) variability^12–17^. Changes in anthropogenic sulfate aerosol forcing has been proposed as the dominant cause of historical multidecadal variations in Atlantic tropical storm frequency based on some Coupled Model Intercomparison Project Phase 5 (CMIP5) model simulations including aerosol indirect effects. In these simulations, a decrease in anthropogenic sulfate aerosol forcing leads to an increase in tropical storms and vice versa^12, 18^. On the other hand, there are large uncertainties in simulated aerosol indirect effects and substantial mean state biases in many CMIP5 model simulations^19, 20^.

In this study, we explore the causes of the recent decline of Atlantic major hurricane frequency over the period 2005–2015, using various observational datasets and modeling results from a 500-year control simulation of a fully coupled earth system model (GFDL-ESM2G). GFDL-ESM2G is a unique CMIP5 model in that its ocean component employs an isopycnal-coordinate^21^, in contrast to most CMIP5 models, which use *z*-coordinate ocean components (“Methods” section). The AMOC structure (especially the depth of AMOC) and the deep overflow in the North Atlantic mean state are far more realistic in isopycnal-coordinate models (such as GFDL-ESM2G) with realistic mixing than in *z*-coordinate models, which have excessive artificial numerical mixing in the deep ocean^22^. The improved AMOC structure and deep overflow in the North Atlantic can greatly reduce the systematic large cold SST bias in the extra-tropical North Atlantic^21, 23^, which is found in most CMIP5 models and often leads to a biased mean state environment for tropical cyclones^20, 24, 25^. Our results suggest an important role of the recent AMOC weakening in the decline of Atlantic major hurricane frequency since 2005. Therefore monitoring and predicting AMOC changes will be important to reduce future Atlantic hurricane risk.

## Results

### Observed coherent relationships

**Fig. 1:**
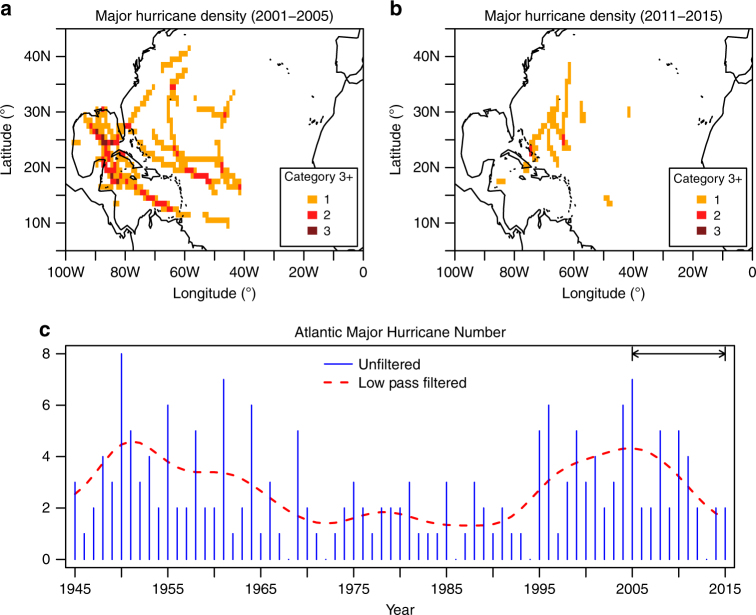
Observed historical Atlantic major hurricane activity. Atlantic major hurricane track density maps are shown for (**a**) 2001–2005; and (**b**) 2011–2015; (**c**) unfiltered Atlantic major hurricane number (blue bar) with the 10-year low-pass filtered time series superimposed (dashed red curve). The key focus period (2005–2015) is highlighted by the line segment/arrows. The track density (color shading) in (**a**, **b**) shows the total number of major hurricanes that occurred at each grid box within each period

**Fig. 2:**
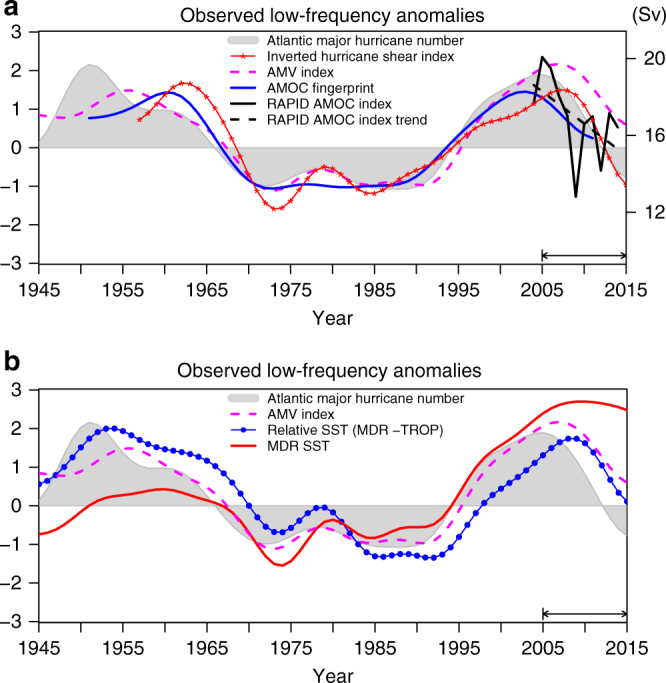
Observed low frequency anomalies in Atlantic major hurricane frequency and associated climate variables. **a** Coherent variations are shown for Atlantic major hurricane frequency (grey shading), Inverted Hurricane Shear Index during the Atlantic hurricane season (JJASON) (solid red with stars), Atlantic Multidecadal Variability (AMV) Index (dashed magenta) and Atlantic Meridional Overturning Circulation (AMOC) Fingerprint (solid blue). The AMOC Fingerprint is shifted backward by 4 years to represent the AMOC anomalies at mid-high latitudes. Also shown are the unfiltered annual mean RAPID AMOC Index at 26°N for 2004–2014 (solid black line) and its linear trend (dashed black line) with a unit of Sverdrup (Sv, right *y*-axis) (**b**) Variations of Atlantic major hurricane frequency (grey shading) and AMV index (dashed magenta) are compared with the main development region (MDR: 80°W–20°W, 10°N–20°N) averaged SST anomalies (solid red) and relative SST (solid blue with dots) during the Atlantic hurricane season (JJASON). The relative SST is defined as the difference between MDR SST anomalies and tropical (TROP: 30°S-30°N) mean SST anomalies. All time series are 10-year low-pass filtered and normalized by their own standard deviation over the period 1957–2005. The key focus period (2005–2015) is highlighted by the line segment/arrows on plot

Figure 1a, b shows contrasting Atlantic major hurricane track density maps from near the peak of the above-normal activity period (2001–2005) and during the less-active recent 5-year period (2011–2015). There is a decline in the Atlantic major hurricane frequency over the period 2005–2015, similar to the rapid decline in the 1960s (Figs 1c and 2a). A similar decline over the period 2005–2015 is also present in the Atlantic hurricane frequency series (Supplementary Fig. 1). This decline is likely related to the reduction of US landfalling hurricanes over 2005–2015. Here we use both observations and model simulations to further explore the causes of the 2005–2015 decline. In particular, we focus on Atlantic major hurricane frequency due to its high societal impact (e.g., Atlantic major hurricanes over the period 1900–2005 are estimated to have contributed ~85% of the total mainland US hurricane damage^1^).

Figure 2a indicates that the observed low-pass filtered Atlantic major hurricane frequency co-varies statistically with the observed AMV Index over the entire period of 1945–2015. The correlation between them is 0.91, which is statistically significant at the 0.05 level ($p < 0.05$). In this study, the AMV Index is defined as the low-pass filtered area-averaged detrended SST anomalies over the mid-high latitude North Atlantic. This averaging region is a key region for the AMV^15, 26^, and the observed SST signal associated with the AMV is strongest there^7, 26^. Since there are no continuous direct observations of historical AMOC variations before 2004^27^, we use a previously identified AMOC fingerprint as a proxy for AMOC variations^28^. The fingerprint, defined as the leading mode of the observed detrended subsurface ocean temperature anomalies at 400 m in the extra-tropical North Atlantic^28^, exhibits a dipole spatial pattern with opposite signed changes in the subpolar gyre and Gulf Stream regions (Supplementary Fig. 2). The AMOC fingerprint is shifted backward in time by 4 years to better represent the AMOC anomalies at mid-high latitudes. The AMOC anomalies at mid-high latitudes lead the fingerprint by ~4 years due to their slow southward propagation^29, 30^. Fig. 2a shows that on multidecadal time scales, the observed AMOC proxy co-varies with both the observed AMV Index and the observed Atlantic major hurricane frequency. The correlation between the AMOC fingerprint and the AMV Index (Atlantic major hurricane frequency) is 0.90(0.92), with $p < 0.05$. In particular, we infer that the decline in the observed AMV Index and Atlantic major hurricane frequency over 2005–2015 are accompanied by an AMOC weakening, as indicated by the observed temporal evolution of the AMOC fingerprint (Fig. 2a) as well as other direct and indirect observations^27, 31, 32^.

The directly observed AMOC Index at 26°N measured from the RAPID Array^27, 33^ (“Methods” section) also exhibits a pronounced decline^27, 31, 32, 34^ that is roughly coincident with the decline in Atlantic major hurricane frequency over the most recent decade (Fig. 2a). The recent observed AMOC decline has been inferred to be part of a decadal/multidecadal AMOC signal^34^, according to a high-resolution data assimilation product. The simulated AMOC increase during the mid 1990s and the AMOC decline over the recent decade from the data assimilation product^34^, as well as the directly observed AMOC decline from the RAPID array data since 2004, are all consistent with our inferences using the AMOC fingerprint and further support the capability of this fingerprint as a proxy for AMOC variations. Despite the evidence cited above from observations and modeling studies^28, 30^, we note that there is inherently less certainty regarding this fingerprint due to the lack of direct observations before 2004.

Previous studies^4, 8, 35^ show that the vertical wind shear over the Atlantic hurricane main development region (MDR, defined here as 80°W–20°W, 10°N–20°N) is strongly correlated with Atlantic major hurricane frequency. Strong vertical wind shear affects storm activity by disrupting the axisymmetric organization of deep convection and hence inhibiting the formation and intensification of Atlantic hurricanes^36^. Figure 2a shows that the observed MDR Hurricane Shear Index (“Methods” section) is strongly anti-correlated with the observed Atlantic major hurricane frequency, AMV Index, and AMOC fingerprint on multidecadal time scales, including the recent declines in these indices. The correlations of the Hurricane Shear Index against the observed Atlantic major hurricane frequency, AMV Index, and AMOC fingerprint are −0.88, −0.85 and −0.92 (all with $p < 0.05$). Decadal shifts similar to the recent decline of major hurricane frequency and the other indices are also seen in the 1960s, along with up swings during the 1990s. A previous study showed that the AMV associated with AMOC variability could induce multidecadal variations in the vertical wind shear over the Atlantic hurricane MDR similar to that observed^8^. Hence the vertical wind shear appears to be a key process by which the AMV/AMOC can modulate Atlantic hurricane activity^4, 8^. At multidecadal time scales over the MDR region, other environmental factors we analyzed from the reanalysis data (e.g., 850 hPa relative vorticity, 700 hPa relative humidity, and 500 hPa vertical velocity) do not show significant correlations with the multidecadal variations of Atlantic major hurricane frequency (Supplementary Fig. 3), even though these factors are important for individual cyclone genesis and intensification^37, 38^. The observed coherent multidecadal variations between the inverted Hurricane Shear Index and Atlantic hurricane/major hurricane frequency suggest that the inverted Hurricane Shear Index can be used as a proxy for the multidecadal variations of Atlantic hurricane/major hurricane frequency.

The coherent variation during the period 2005–2015 of Atlantic major hurricane frequency, AMV Index, AMOC Index/fingerprint, and inverted Hurricane Shear Index provides new evidence from several independent observational sources for an important role for AMOC in the observed recent decline of the Atlantic major hurricane frequency. Atlantic hurricane frequency exhibits similar coherent multidecadal variability to that of Atlantic major hurricane frequency (Supplementary Fig. 1c and Supplementary Fig. 4). These variations in category 1–5 hurricane frequency are also statistically coherent with the AMV Index, AMOC fingerprint, and inverted Hurricane Shear Index (Supplementary Fig. 4).

On multidecadal time scales, local MDR SST also exhibits similar variations to the Atlantic major hurricane frequency except that the MDR SST has a pronounced increasing trend over the entire period, in contrast to hurricane frequency, which has little trend. Major hurricane frequency is consistently related to relative SST^5^ (difference between MDR SST and tropical mean SST), as they are correlated while neither index has an obvious trend (Fig. 2b). Interestingly, at low frequency, relative SST lags behind major hurricane frequency by a few years (Fig. 2b and Supplementary Fig. 5). Although one must treat lagged correlations of low-pass filtered time series with caution^39^, this result suggests a possibly important role for the extra-tropical North Atlantic ocean in modulating Atlantic major hurricane frequency at the multidecadal time scale, perhaps by remote influence on vertical shear through an atmospheric bridge^40, 41^. This interpretation is also consistent with studies suggesting that tropical SST responds, with some lag, to extra-tropical North Atlantic oceanic changes at low frequency through oceanic and atmospheric teleconnections^15, 26, 40^. Further, initialization of the extra-tropical, but not tropical, North Atlantic ocean state, was shown to be crucial for multi-year predictability of Atlantic MDR vertical wind shear and tropical storm numbers^41^.

**Fig. 3:**
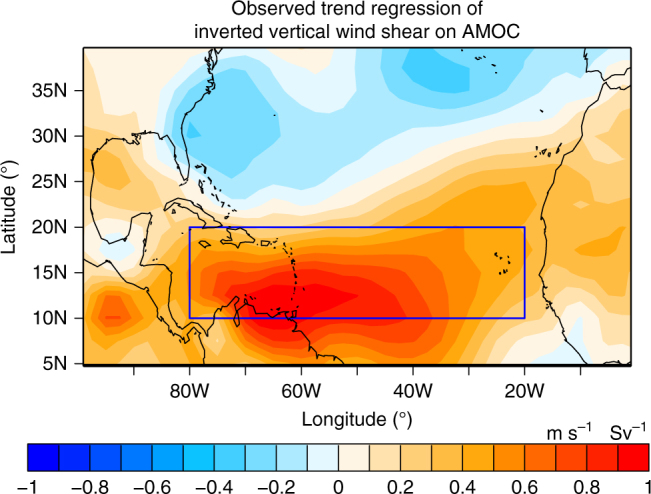
Observed trend regression of inverted vertical wind shear on the AMOC Index. The 11-year trends of inverted vertical shear of zonal wind are regressed on the 11-year trends of AMOC Index at 26°N. The observed AMOC Index at 26°N is reconstructed using the AMOC fingerprint (Methods). Blue box: Atlantic hurricane main development region (MDR: 80°W–20°W, 10°N–20°N). The results are based on all available 11-year segments sampled from the observations. Positive values imply an increase in vertical wind shear when AMOC declines, and vice versa

To test whether the observed relationship between the declining trend in the AMOC Index at 26°N and the increasing trend in the Hurricane Shear Index over the recent period 2005–2015 is a robust statistical relationship, we sampled all available 11-year segments from observations since 1957 to derive a regression map of 11-year trend of inverted vertical shear of zonal wind versus 11-year trend of reconstructed AMOC Index at 26°N (Fig. 3). The observed AMOC Index at 26°N is reconstructed from the AMOC fingerprint (“Methods" section). Figure 3 shows that the observed MDR vertical shear of zonal wind is robustly anti-correlated with the AMOC Index at 26°N. This provides additional evidence that the rapid increase of Hurricane Shear Index over the period 2005–2015 is statistically related to the AMOC decline.

### Model simulated coherent relationships

To further explain this relationship, we analyze results from the GFDL-ESM2G control simulation. In this simulation, the spatial pattern of the model’s AMOC fingerprint is similar to that observed, which includes a dipole pattern with opposite signed changes in the subpolar gyre and the Gulf Stream regions (Supplementary Fig. 6). The model’s AMOC fingerprint lags its AMOC Index at 42**°**N by ~4 years, with a maximum correlation of 0.93 ($p < 0.01$). Similarly, the model’s AMOC fingerprint lags its AMOC Index at 26°N by ~1 year, with a maximum correlation of 0.90 ($p < 0.01$). The simulated coherent multidecadal variations between the AMOC fingerprint and the AMOC Index at 26°N are consistent with the observed coherent decline in the AMOC fingerprint and the AMOC Index at 26°N over the recent decade (Fig. 2a) and consistent with a previous study^28^. This confirms, within the model climate, the capability of using the model’s AMOC fingerprint as a proxy for its AMOC variations, and further supports the use of this approach in observations.

**Fig. 4:**
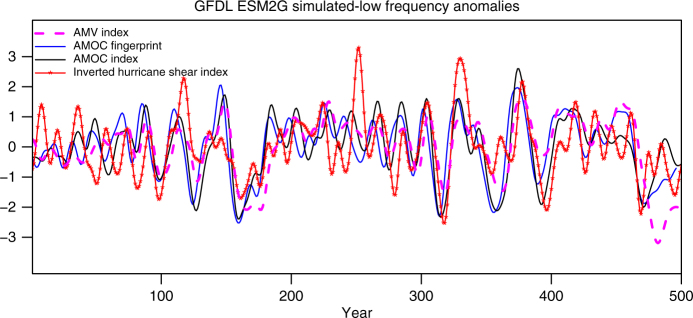
Modeled low frequency anomalies from the GFDL-ESM2G control simulation. Coherent variations are shown for the Atlantic Multidecadal Variability (AMV) Index (dashed magenta), the Atlantic Meridional Overturning Circulation (AMOC) Fingerprint (solid blue), the AMOC Index at 26°N (solid black) and the Inverted Hurricane Shear Index during the Atlantic hurricane season (JJASON) (solid red with stars). The AMOC Fingerprint is shifted backward by 4 years to represent AMOC anomalies at mid-high latitudes. All time series are 10-year low-pass filtered and normalized by their own standard deviation over the entire period

In the GFDL-ESM2G control simulation, the AMV Index has a maximum correlation of 0.72 ($p < 0.01$) with the AMOC Index at 26°N when the AMOC Index leads by about 2 years. The simulated coherence among the AMV Index, low-pass filtered AMOC fingerprint and AMOC Index (Fig. 4) is consistent with the observed coherent decline in the AMV Index, AMOC fingerprint, and the RAPID AMOC Index at 26°N in the recent decade (Fig. 2a) and further suggests that ocean dynamics has played an important role in the AMV^15, 17, 28, 32, 42–49^. The simulated low-pass filtered inverted MDR Hurricane Shear Index is also significantly correlated with the AMV at zero lag (r = 0.48, $p < 0.01$), and with the low-pass filtered AMOC Index at 26°N when the AMOC Index leads by about 2 years (*r* = 0.59; $p < 0.01$) (Fig. 4). This provides additional model-based evidence that the recent increase in the Hurricane Shear Index and decline of Atlantic major hurricane frequency could be induced by the recent AMOC weakening (Fig. 2a).

**Fig. 5:**
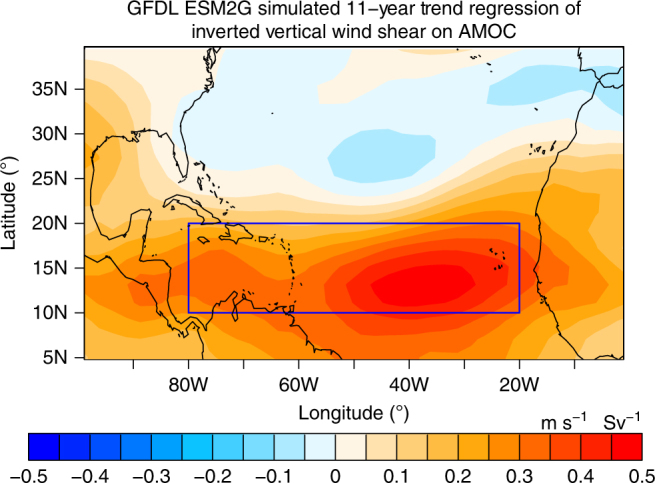
Modeled trend regression from the GFDL-ESM2G control simulation. The 11-year trends of inverted vertical shear of zonal wind are regressed on the 11-year trends of the AMOC Index at 26°N. Blue box: Atlantic hurricane main development region (MDR: 80°W–20°W, 10°N–20°N). The results are based on all available 11-year segments sampled from the control simulation. Positive values imply an increase in vertical wind shear when AMOC declines, and vice versa

Figure 5 shows the regression of 11-year trends of the model’s inverted vertical shear of zonal wind against 11-year trends of its AMOC Index at 26°N, based on all available 11-year segments sampled in the GFDL-ESM2G control simulation. Similar to the observed results (Fig. 3), the simulated MDR vertical shear of zonal wind increases with a decline in the AMOC Index at 26°N and vice versa. The simulated regression has an eastward shift in its maximum and a smaller amplitude than that observed (Figs 3 and 5), which we speculate is probably related to model mean state biases in the tropical Atlantic^50^. Another possible factor for this model deficiency is that the simulated AMOC Index/fingerprint has widely varying amplitude of multidecadal variations over the 500-year simulation (Fig. 4). For example, during the first 100 years and year 200–300, the simulated AMOC variability is relatively smaller, has shorter typical periods, and its connection with the Hurricane Shear Index is weaker, compared with other segments of the control simulation with more pronounced multidecadal AMOC variations (Fig. 4). This suggests that in the real world, the connection of Atlantic major hurricane frequency and AMOC might vary on centennial time scales and may depend on the amplitude and time scale of AMOC variability, although the observational record is too short to confirm this.

The results from the GFDL-ESM2G control simulation demonstrate that the observed statistical relationships among the AMOC Index, AMOC fingerprint, AMV Index, and MDR vertical shear of zonal wind at decadal/multidecadal time scales also exist in this coupled model (Figs 4 and 5). These modeling results of the teleconnections linked to AMOC changes (including the dynamical response of vertical shear of zonal wind over the MDR region regressed on the AMOC Index) provide more evidence and further support our hypothesis that AMOC variations are important for the recent observed decline in the Atlantic major hurricane frequency.

### Recent changes of anthropogenic aerosol optical depth

**Fig. 6:**
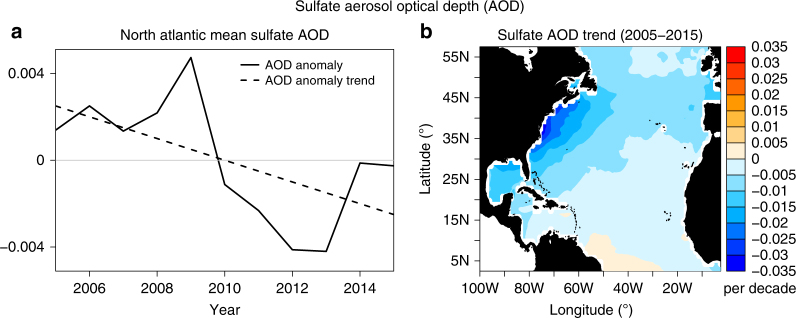
Observed changes of anthropogenic sulfate aerosol optical depth. **a** The area-averaged sulfate aerosol optical depth (AOD) anomaly (solid black line) over the North Atlantic (80°W–20°W, 0°–60°N) and its linear trend (dashed black line) are shown for the period 2005–2015. **b** Sulfate AOD trends over the North Atlantic region. The sulfate AOD results are derived from the Modern Era Retrospective analysis for Research and Applications Aerosol Reanalysis (MERRAero)^64^

Anthropogenic aerosols have been proposed as a dominant causal mechanism for AMV^12^ and multidecadal Atlantic tropical cyclone variability^18^, with increasing (decreasing) aerosol loadings leading to decreased (increased) SST and tropical cyclone frequency. Recent observations indicate that anthropogenic aerosol emissions and optical depth over the North Atlantic region have decreased during the early 21st century^51–54^. Figure 6a shows the observed area-averaged anthropogenic sulfate aerosol optical depth (AOD) anomalies in the North Atlantic for the period 2005–2015. There is a slight declining trend in sulfate AOD over this period. A spatial map of sulfate AOD trend over this period shows widespread declining trends over most of the North Atlantic region (Fig. 6b). Under the proposed mechanisms^12, 18^, declining sulfate AOD would be expected to allow more shortwave radiation to reach the surface, leading to North Atlantic surface warming that favors tropical storm and hurricane formation. However, the observed decline in the AMV Index and the Atlantic major hurricane frequency during 2005–2015 suggest that changes in anthropogenic aerosols were not likely the primary driver of AMV and Atlantic major hurricane activity changes over this period.

## Discussion

Our analysis of various observations and a GFDL-ESM2G control simulation collectively suggest an important role of the AMOC in the recent decline of the Atlantic major hurricane frequency during 2005–2015. Directly observed North Atlantic sulfate aerosol optical depth has not increased (and instead has shown a modest decline) over this period. Thus the decline of Atlantic major hurricane frequency during 2005–2015 is not likely due to recent changes in anthropogenic sulfate aerosols. We expect, based on our findings, that changes in the Atlantic major hurricane frequency in the next decade would be closely linked to future AMOC changes. Besides, a basin-wide decline in the Atlantic hurricane frequency induced by AMOC weakening could be related to the rapid intensification of the US landfall hurricanes^55^. Consequently, we propose that monitoring and predicting AMOC changes will be crucial for developing a robust predictive understanding of future Atlantic hurricane risk.

## Methods

### Observational data

The observed Atlantic major hurricane frequency data were obtained from the Atlantic basin hurricane database (HURDAT), with no bias corrections applied in this case^56, 57^. The original unfiltered observed Atlantic major hurricane (hurricane) series is shown in Fig. 1c (Supplementary Fig. 1c). In Supplementary Fig. 1c, the observed Atlantic hurricane frequency during the period 1945–1965 is adjusted by a correction for estimated missing storms as inferred from ship track density during these years^5^. To construct the observed Atlantic hurricane/major hurricane track density maps (Fig. 1), the Best track data (HURDAT2)^58^ is interpolated to a temporal resolution of 1 h.

The HadISST1.1 data set^59^ is used for the calculation of the observed AMV index. In this study, the AMV Index is defined as the detrended (trend begins in 1900) annual mean area-averaged SST in the mid-high latitude North Atlantic (60°W–0°, 50°N–65°N). The time series of the ‘AMOC fingerprint’ is defined as the leading principal component (PC1) of detrended subsurface ocean temperature anomalies at 400 m in the extra-tropical North Atlantic (80°W–0°, 20°N–65°N)^28^. Using upper ocean heat content gives very similar results to 400 m temperature. The AMOC fingerprint is shifted 4-year backward in Figs 2 and 4 to represent AMOC anomalies at mid-high latitudes, which systematically lead the AMOC fingerprint by ~4 years due to a slow southward propagation of AMOC anomalies^29, 30^. The observed ‘AMOC fingerprint’ (Supplementary Fig. 2) is derived from an objectively analyzed data set of annual mean ocean temperature anomalies^60^. The AMOC Index at each latitude is defined as the maximum of annual mean Atlantic meridional overturning streamfunction at that latitude.

The directly observed AMOC Index at 26°N, available for the period 2004–2014, is obtained from the RAPID-WATCH MOC monitoring project^27, 33^. To maximize the number of available years in the short data record and be consistent with previous studies^27, 33^, each annual mean RAPID AMOC Index value is for a 12-month period starting on April 1 of the named year and ending on March 31 of the following year. The method of calculating the annual average does not affect the rapid decline trend in RAPID AMOC Index. The RAPID AMOC Index at 26°N during 2004–2014 and its linear trend are superimposed in Fig. 2a. The mean/trend of the observed AMOC Index at 26°N matches that of 1-year backward shifted AMOC fingerprint over their overlapped period of 2004–2014. The 1-year shift is based on the time lag between the AMOC Index at 26°N and AMOC fingerprint in the coupled earth system model (GFDL-ESM2G) control simulation (described below). The observed AMOC Index at 26°N used in Fig. 3 is reconstructed from the observed 1-year backward shifted AMOC fingerprint, and calibrated by the ratio between the trend of the RAPID AMOC Index at 26°N and the trend of the observed 1-year backward shifted AMOC fingerprint over their overlapped period of 2004–2014.

The ERA-Interim^61^ and the ERA-40^62^ atmospheric reanalysis data are used to calculate the observed anomalous 200–850 hPa vertical shear of the zonal wind during the Atlantic hurricane season of June through November (JJASON). The Hurricane Shear Index is defined as the area-averaged 200–850 hPa vertical shear of hurricane seasonal-mean (JJASON) zonal wind over the MDR (80°W–20°W, 10°N–20°N). The data from ERA-Interim^61^ (1979–2015) and ERA-40^62^ (1957–1978) are combined to calculate the observed Hurricane Shear Index for the full period. An alternative Hurricane Shear Index calculated from the NCEP/NCAR Reanalysis^63^ shows similar multidecadal variations over the same period (Supplementary Fig. 7). Note the pre-1960 vertical wind shear data are likely to have relatively larger uncertainties. The relative SST Index^5^ is defined as the difference between SST anomalies averaged over the MDR (MDR SST) and tropical (30°S-30°N) mean SST anomalies (TROP SST) during the Atlantic hurricane season (JJASON). The anomalies and trend pattern of sulfate AOD at 550 nm in North Atlantic are calculated from the monthly mean Modern Era Retrospective analysis for Research and Applications Aerosol Reanalysis (MERRAero)^64^.

### Model description

Model results are calculated from a 500-year pre-industrial control simulation of the Geophysics Fluid Dynamics Laboratory (GFDL) earth system model ESM2G^21^. The ocean component of GFDL-ESM2G uses the isopycnal-coordinate with 64 vertical layers, and 1° horizontal resolution with the meridional resolution refined to 1/3° at the equator. The atmospheric component of GFDL-ESM2G has 24 vertical levels, with horizontal resolution of 2° × 2.5°. The control simulation uses 1860 constant radiative forcing conditions.

### Statistical processing

All the low-pass filtered data are obtained using the R function “filtfilt”, with a Hamming window based low-pass filter and a frequency response that drops to 50% at a 10-year cutoff period. Padding points for low-pass filtering are derived by mirroring the data at each end (i.e., zero slope at endpoints). The observed anomalies are referenced to the corresponding climatological means of a common period of 1957–2005. The statistical significance for the correlations between a pair of low-pass filtered time series discussed in the paper are assessed using a Monte Carlo simulation based on a random phase method of resampling^65^.

### Code availability

The data involved in this study are analyzed by using the widely available analysis tools R and Ferret. Contact X.Y. or R.Z. for specific code requests.

### Data availability

Websites of the data used in this study:

HURDAT: http://www.aoml.noaa.gov/hrd/hurdat/comparison_table.html

HURDAT2: http://www.nhc.noaa.gov/data/#hurdat

HadISST1.1: http://www.metoffice.gov.uk/hadobs/hadisst/data/download.html

Subsurface oceanic temperature anomalies: https://www.nodc.noaa.gov/OC5/3M_HEAT_CONTENT/

ERA-Interim, ERA-40 data: http://apps.ecmwf.int/datasets/

NCEP/NCAR Reanalysis: http://www.esrl.noaa.gov/psd/data/gridded/data.ncep.reanalysis.derived.surface.html

RAPID AMOC Index: www.rapid.ac.uk/rapidmoc

Adjusted Hurricane number in Vecchi and Knutson, 2011, J. Climate:

https://www.gfdl.noaa.gov/wp-content/uploads/files/user_files/gav/historical_storms/vk_11_hurricane_counts.txt

GFDL-ESM2G model data: http://nomads.gfdl.noaa.gov:8080/DataPortal/cmip5.jsp

## Electronic supplementary material

Supplementary Information
