# Supplementary material for: The role of Atlantic overturning circulation in the recent decline of Atlantic major hurricane frequency
Source: Nat Commun. 2017 Nov 22;8:1695. doi: 10.1038/s41467-017-01377-8 (PMC5700105; doi:10.1038/s41467-017-01377-8)
Supplement: Supplementary file 1 — Supplementary Information [file 41467_2017_1377_MOESM1_ESM.pdf]

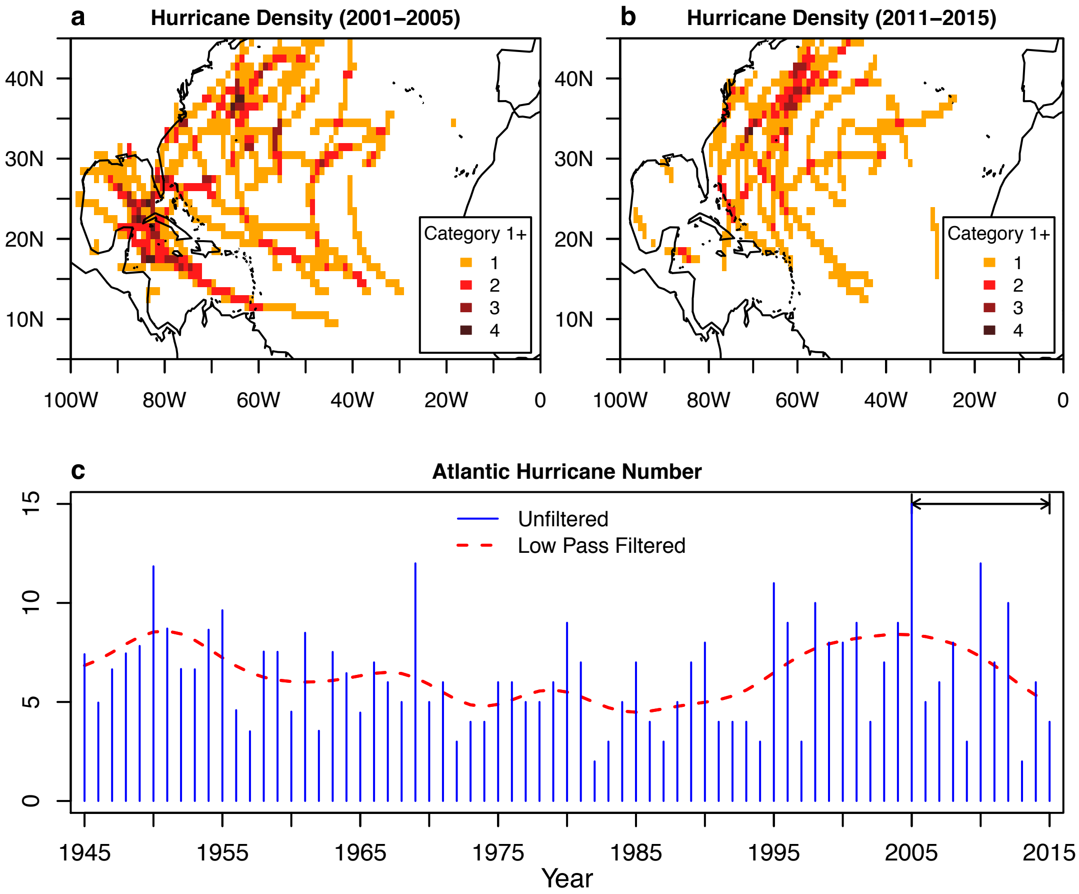

Supplementary Figure 1: Observed historical Atlantic hurricane activity. Atlantic hurricane track density maps are shown for **a**) 2001-2005; and **b**) 2011-2015; **c**) unfiltered Atlantic hurricane number (blue bar) with the 10-yr low-pass filtered time series superimposed (dashed red curve). The key focus period (2005-2015) is highlighted by the line segment/arrows. The track density (color shading) in **a**), **b**) shows the total number of Atlantic hurricanes that occurred at each grid box within each period. The hurricane counts from 1945 to 1965 are adjusted for missing storms using observing ship track density[1].

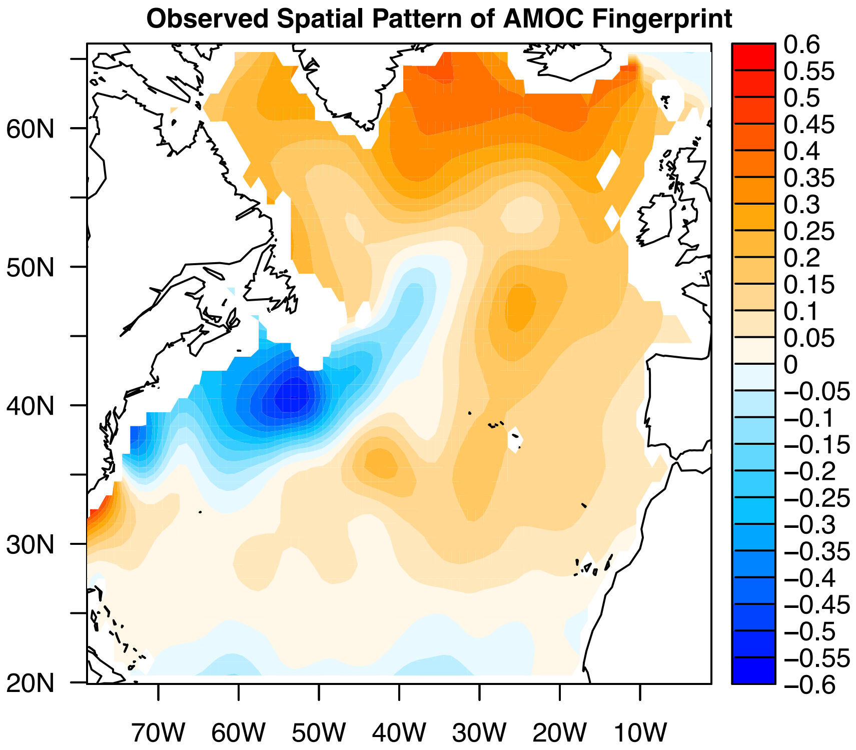

Supplementary Figure 2: Spatial pattern of observed Atlantic Meridional Overturning Circulation fingerprint. The Atlantic Meridional Overturning Circulation (AMOC) fingerprint is defined as the first EOF of detrended subsurface ocean temperature anomalies (K) at 400m in the extra-tropical North Atlantic. Note that using upper ocean heat content gives very similar results as 400m temperature. The data are derived from an objectively analyzed dataset of annual mean ocean temperature anomalies (Methods).

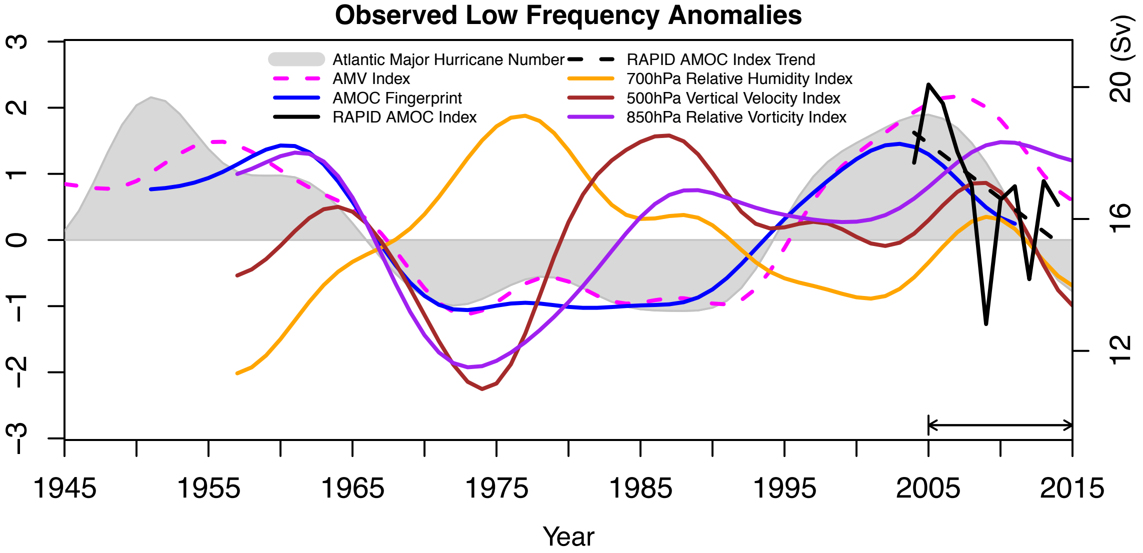

Supplementary Figure 3: Observed low frequency anomalies of Atlantic major hurricane number and associated environmental indices. Same as Figure 2**a**, but replacing inverted Hurricane Shear Index with indices of other environmental factors (700hPa Relative Humidity Index, 500hPa Vertical Velocity Index, and 850 Relative Vorticity Index), which are calculated from the ERA-Interim and ERA-40 data. The key focus period of our study (2005-2015) is highlighted by the line segment/arrows.

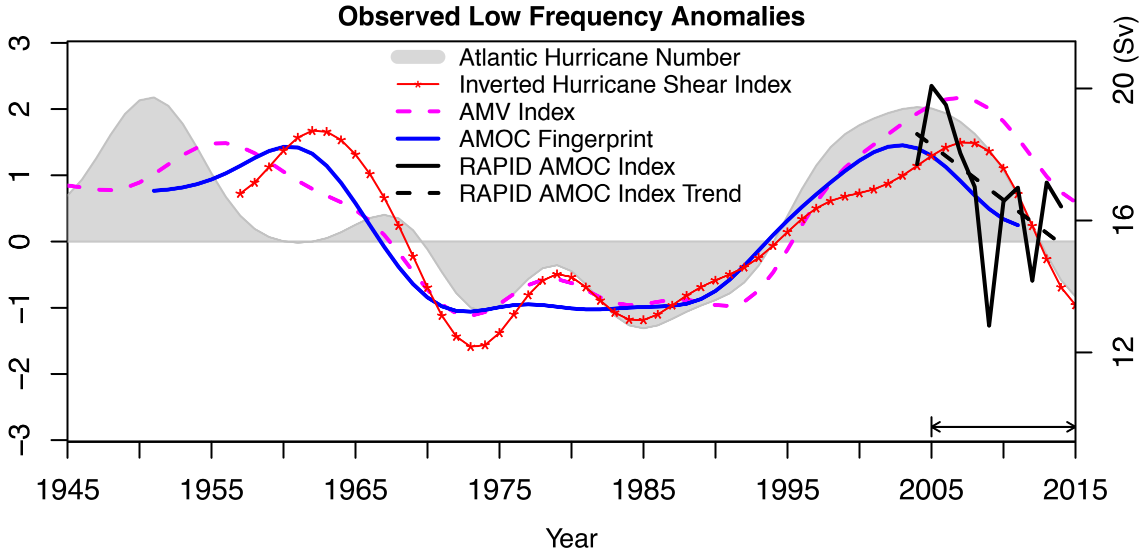

Supplementary Figure 4: Observed low frequency anomalies in Atlantic hurricane number and associated climate variables. Same as Figure 2**a**, but for the Atlantic hurricane (category 1-5) number. The key focus period (2005-2015) is highlighted by the line segment/arrows.

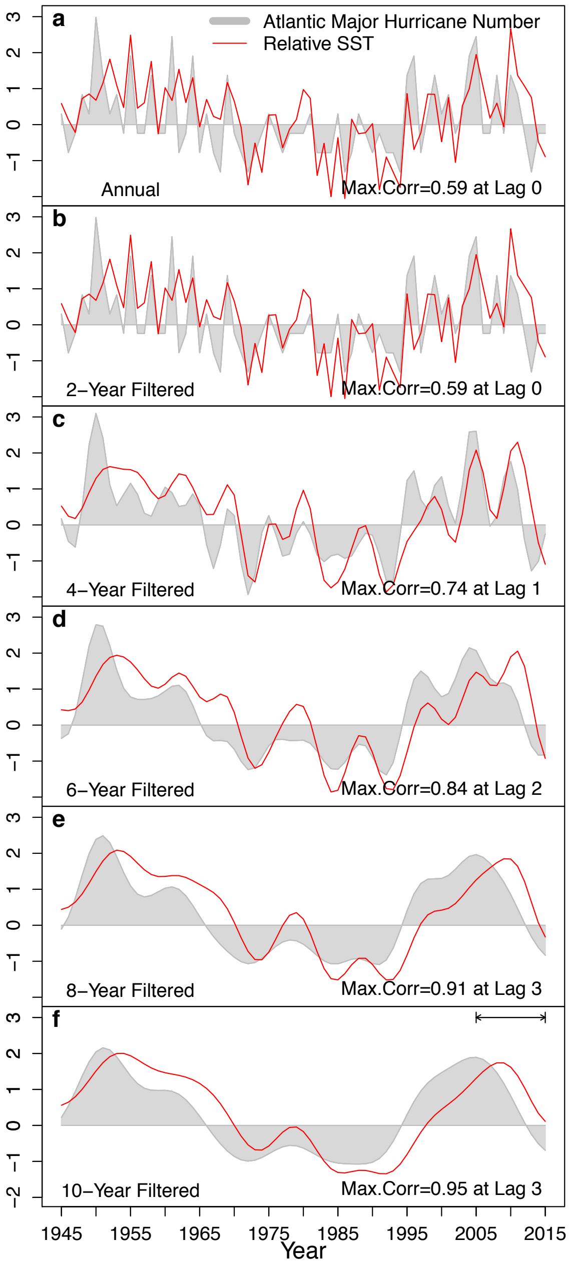

Supplementary Figure 5: Observed time series of Atlantic major hurricane frequency and relative SST. a) unfiltered; **b**) 2-yr low-pass filtered; **c**) 4-yr low-pass filtered; **d**) 6-yr low-pass filtered; **e**) 8-yr low-pass filtered; **f**) 10-yr low-pass filtered. The relative SST is defined as the difference between the main development region (MDR: 80ºW-20ºW, 10ºN-20ºN) SST anomalies and tropical (TROP: 30ºS-30ºN) mean SST anomalies during the Atlantic hurricane season. The key focus period (2005-2015) is highlighted by the line segment/arrows on **f**).

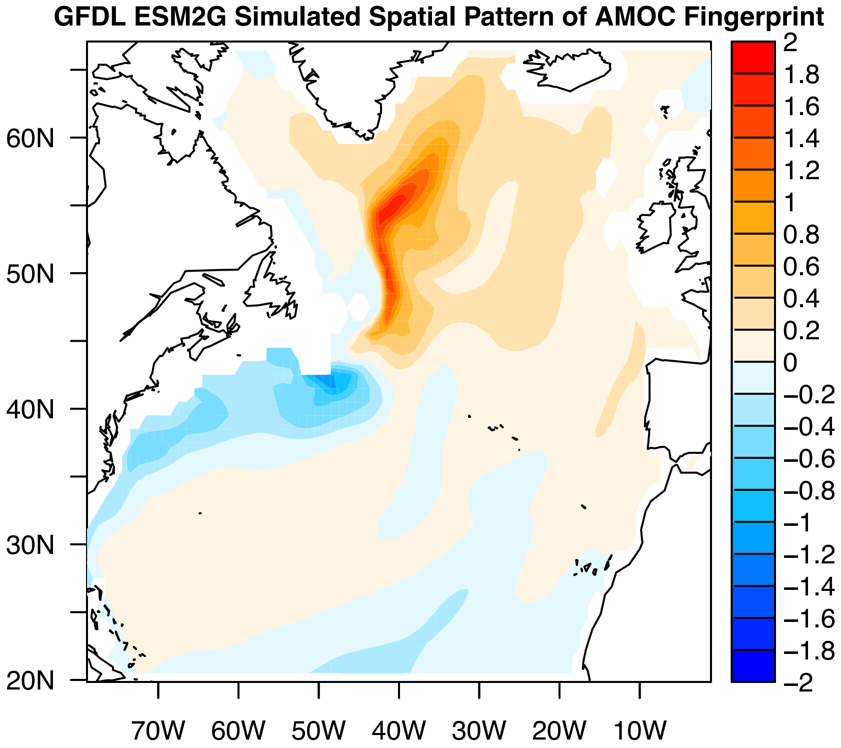

Supplementary Figure 6: Spatial pattern of the Atlantic Meridional Overturning Circulation fingerprint in the GFDL-ESM2G 500-year pre-industrial control simulation. The Atlantic Meridional Overturning Circulation (AMOC) Fingerprint is defined as the first EOF of detrended subsurface ocean temperature anomalies (K) at 400m in the extra-tropical North Atlantic. Note using upper ocean heat content gives very similar results as 400m temperature.

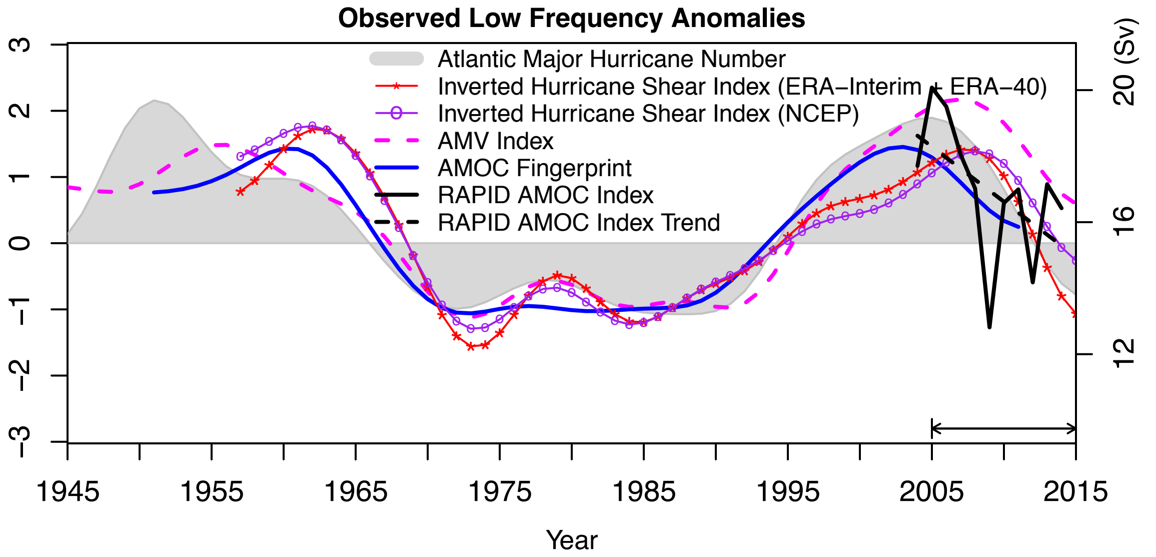

Supplementary Figure 7: Observed low frequency anomalies in Atlantic hurricane number and associated climate variables. Same as Figure 2**a**, but including a comparison of a Hurricane Shear Index calculated from NCEP/NCAR Reanalysis with that calculated from the ERA-Interim and ERA-40 data. The linear trend over the period 1957-2015 is removed from both Hurricane Shear Indices. The key focus period of our study (2005-2015) is highlighted by the line segment/arrows.

Supplementary references

1. Vecchi, G.A. & Knutson, T.R. Estimating annual numbers of Atlantic hurricanes missing from the HURDAT database (1878-1965) using ship track density. *J. Climate*, **24**, 1736-1746 (2011).
